# Complex Nature of Hominin Dispersals: Ecogeographical and Climatic Evidence for Pre-Contact Craniofacial Variation

**DOI:** 10.1038/s41598-019-48205-1

**Published:** 2019-08-13

**Authors:** Ann H. Ross, Douglas H. Ubelaker

**Affiliations:** 1 North Carolina State University, Department of Biological Sciences, Raleigh, 27695 United States; 2 Smithsonian Institution, National Museum of Natural History, Washington, DC 20560 United States

**Keywords:** Biological anthropology, Phylogenetics

## Abstract

Coordinate data analysis of ancient crania from the New World reveals complexity in interpretation when addressing ancient population dispersals. The results of this study generally support a geographic patterning for the New World; however, it also revealed a much more complex and multifactorial mechanism shaping craniofacial morphology that should be considered when investigating ecogeographic models for hominin dispersals. We show that craniofacial variation is not the result of a single mechanism but is a much more complex interaction of environmental and microevolutionary forces.

## Introduction

The dispersal of the human lineage from African origins to a world-wide presence has been the focus of intense interest, particularly when and how humans came to occupy the New World has been the subject of multiple studies^1,2^. Climatic, dietary changes, and altitude have been proposed as factors resulting in human diversification, indicated by measurable differences in craniofacial variation reflecting human dispersal patterns^3,4^.

For decades, scholarly anthropological interest has focused on the antiquity of the human presence in the New World. Discoveries in South America have provided key information needed to understand both the timing and pattern of demic expansions into the New World. Much of this evidence from Meso- and South America, is based on archaeological data, which has led to both debated and accepted hypotheses relating to chronology^5–17^ and demic diffusion^18,19^. Climate has been found to be a major driver in human migrations and subsequent diversification^3,4^. For example, the last glaciation^20^, which occurred 30–13 kya, opened an ice-free corridor allowing the first migrations from Siberia.

Linguistic classification^21^, along with archaeological and biological data, have contributed to hypotheses and interpretations related to origins^22–25^, migration routes^26^, and the general number of migrations involved^27–30^ for the peopling of the New World. Although genetic (mtDNA) studies agree on an Asian origin for the five Native American mtDNA haplotypes (A, B, C, D, and X)^30–33^, the absence of B and X in northern Asia and North America fails to support a single founding migration hypothesis^33^. Furthermore, it has been argued that modern genomic studies reflect later migrations from the south and not the original founding population that was depopulated in the northern latitudes on all continents during the last glaciation^33^. An alternative hypothesis suggests a Central Asian rather than a Northern Asian migration that did not leave an admixture trail^31^. Others argue for a coastal Pacific migration based on the coastal distribution of the rare D-subtype D4h3 found in North and South America and stress that human dispersals were more complex than genetic studies of modern populations suggest^34,35^. However, these genetic studies are not without bias as many lump available ancient skeletal samples into a single sample with a large proportion of these genetic modellings being based on extant populations and not ancient ones^36^.

The need for a general study of the nature of craniofacial variation has been noted for some time^37^ and broad synthetic studies of Pre-Columbian craniofacial variation are required to better understand morphological patterns and to establish a comparative foundation with which to assess individual discoveries, especially those with considerable antiquity. However, before conclusions or interpretations can be made with respect to early Paleoamerican variation and/or migration models, or the effects of European and African populations on modern Latin American populations, a baseline of craniofacial variation present before European contact and after the period of major Native American population migrations into the New World must be established.

Here, we propose multifactorial evidence for Pre-Contact craniofacial morphological variation that supports more than one mechanism influencing morphology, which can be used as a baseline for future studies.

## Results

**Figure 1:**
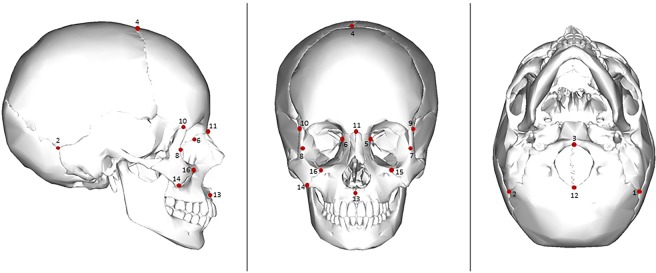
Sixteen homologous anatomical landmarks used in this study (image adapted from; https://commons.wikimedia.org/wiki/File:Human_skull_-_anterior_view.png**;** https://commons.wikimedia.org/wiki/File:Human_skull_-_inferior_view.png**;** https://creativecommons.org/licenses/by-sa/3.0/deed.en**)**.

A complex mechanism for pre-contact New World craniofacial variation is revealed showing multifactorial forces from spatial/geographic distribution, altitude, and climate, as well as demic diffusion and drift modeling morphology in a sample of 257 individuals from 10 localities using 16 homologous anatomical landmarks (Fig. 1, Tables 1, 2, see Materials and Methods section; the raw coordinate data used in this study are available in the Supplementary file).

**Table 1:** Sample Composition, elevation, latitude/longitude, chronology, climate, and provenance.

Sample	N	Elevation (in meters)	Latitude	Longitude	Chronology	Climate	Sample Location
Chile North	13	4331.4	−19.9833	−69.7833	AD800–1200 Highland	Hot arid	Universidad de Chile, Santiago Chile
Chile South	10	546.8	−46.5076	−74.1256	AD1400–1630	Wet oceanic	Universidad de Chile, Santiago Chile
Ecuador	23	18	−2.36	−80.166	AD730–1500 Coastal	Subtropical	NMNH, Smithsonian Institution Washington DC
Mexico Total	49						
*Chihuahua*	13	1431.7	28.6353	−106.089	Tarahumara Culture Unknown	Cool temperate xeric	Peabody Museum, Harvard, MA
*Chichen-Itza*	4	40	20.6828	−88.57	Prehistoric Mayan Unknown	Subtropical	Peabody Museum, Harvard, MA
*Michoacan*	31	63	22.3333	−103.05	Prehistoric Tarasco Unknown	Subtropical	Carl Lumholtz Collection, American Museum of Natural History, New York, NY
Northern South America	9	1249	13.5915	−72.5334	Pre-Contact Unknown	Subtropical	NMNH Smithsonian Institution Washington DC
Panama	6	294.4	8.15	−81.4	Pre-Contact Unknown	Subtropical	Patronato Panama Viejo, Panama
Patagonia Chubut/Rio Negro	23	1093	−41.8102	−68.9063	Pre-Contact, Unknown	Boreal/Alpine	NMNH Smithsonian Institution Washington DC
Peru Total	125						
*Ancon*	47	159	−11.7334	−77.15	AD900–1300 Coastal	Warm temperate xeric	Museo Nacional de Arqueología, Antropología e Historia, Lima Peru
*Cajamarca*	29	2438.4	−6.45497	−78.8383	AD1–1200 Highland	Cool temperate moist	Museo Nacional de Arqueología, Antropología e Historia, Lima Peru
*Maka-Tampu*	49	18.3	−12.0655	−77.1256	AD1–800 Coastal	Warm temperate xeric	Museo Nacional de Arqueología, Antropología e Historia, Lima Peru

**Table 2:** Coordinate landmarks used in the analyses.

1. Left asterion	9. Left frontomalare temporale
2. Right asterion	10. Right frontomalare temporale
3. Basion	11. Nasion
4. Bregma	12. Opisthion
5. Left dacryon	13. Subspinale
6. Right dacryon	14. Right zygomaxillare
7. Left ectoconchion	15. Left zygoorbitale
8. Right ectoconchion	16. Right zygoorbitale

### Geometric morphometrics

The Procrustes ANOVA results show significant group variation for shape (F (451, 10004) = 20.84, p = <0.0001), but did not show significant variation for centroid size (F (11, 244) = 4.88, p = 0.340). The canonical variates analysis (CVA) procedure identified eleven significant canonical axes. The eigenvalues indicate that approximately 72% of the shape variation is accounted for on the first canonical axis and 12% on the second canonical axis for 84% of the shape variation. Table 3 presents the Procrustes distances, which are the distance between the mean shape of each group.

**Table 3:** Procrustes distances showing the absolute shape difference between the groups. Distances are on the lower diagonal and the p-values are on the upper diagonal (numbers in bold depict groups that are not significantly different from each other).

Ancon	Cajamarca	Chichen-Itza	Chihuahua	Ecuador	MakaTampu	Michoacán	N Chile	NSA	Panama	Patagonia	S Chile	
0	<0.0001	0.0010	<0.0001	0.0020	0.0025	<0.0001	0.0043	0.0002	<0.0001	<0.0001	0.0005	Ancon
0.0213	0	<0.0001	<0.0001	0.0092	<0.0001	<0.0001	0.0075	<0.0001	<0.0001	<0.0001	<0.0001	Cajamarca
0.0438	0.0444	0	**0.1253**	**0.2587**	<0.0001	<0.0001	0.0002	**0.6012**	**0.3186**	**0.1470**	**0.4247**	Chichen-Itza
0.0427	0.0438	0.0565	0	0.0114	<0.0001	<0.0001	<0.0001	0.0537	0.0015	0.0004	0.0464	Chihuahua
0.0295	0.0298	0.0455	0.0497	0	<0.0001	<0.0001	**0.4150**	**0.4481**	0.0330	0.0530	**0.0813**	Ecuador
0.0149	0.0260	0.0524	0.0423	0.0337	0	<0.0001	<0.0001	<0.0001	<0.0001	<0.0001	0.0005	MakaTampu
0.4176	0.4156	0.4182	0.4147	0.4157	0.4225	0	<0.0001	<0.0001	<0.0001	<0.0001	<0.0001	Michoacán
0.0226	0.0222	0.0480	0.0485	0.0312	0.0287	0.4128	0	0.0019	<0.0001	0.0045	0.0006	North Chile
0.0323	0.0356	0.0299	0.0466	0.0312	0.0404	0.75	0.0360	0	<0.0001	**0.1215**	**0.1688**	North SA
0.1113	0.1077	0.1159	0.1157	0.1087	0.1148	0.3788	0.1053	0.1111	0	0.0004	0.0016	Panama
0.0312	0.0328	0.0650	0.0481	0.0388	0.0296	0.4246	0.0416	0.0460	0.1133	0	0.0443	Patagonia
0.0585	0.0674	0.0730	0.0667	0.0661	0.0597	0.4344	0.0661	0.0644	0.1312	0.0691	0	South Chile

Based on the Procrustes distances, the Peruvian samples (two coastal and one highland) are significantly different from all other groups and are closest to each other. The Chichen-Itza sample from the Yucatan Peninsula is not significantly different from the sample from Ecuador (also a coastal sample), Northern South America (consisting of a combined sample from Colombia and Venezuela), Patagonia, Southern Chile, Panama, or Mexico (from Chihuahua) samples. The Northern South American sample is not significantly different from either the Southern Chilean or Patagonian samples. The Mexican sample from Chihuahua is significantly different from all other samples, while the sample from Ecuador does not differ significantly from the Northern Chilean, Northern South American, or Southern Chilean samples. The sample from Michoacán is the most dissimilar from all of the samples.

**Figure 2:**
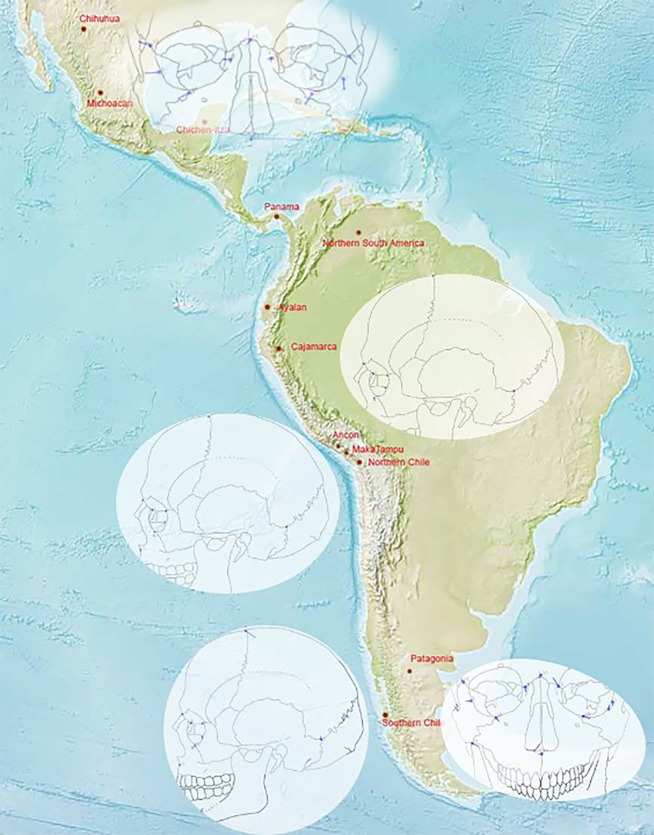
Map showing location of the samples based on latitude and longitude. Difference vector plots show the direction and magnitude of shape change and are presented for the Peruvian highland (Cajamarca) sample deformed into the Northern Chilean sample, for the coastal Peruvian samples, for the Yucatan Chichen-Itza sample deformed into the coastal sample from Ecuador, for the Southern Chilean sample deformed into the Patagonian sample, and for two Mexican samples (Chichen-Itza and Michoacán). Map adapted from https://www.cia.gov/library/publications/the-world-factbook/attachments/images/large/world_phy.jpg?1558019809.

Morphological variation is graphically illustrated via difference vectors (blue lollipops) that depict the direction and magnitude of shape change for the anatomical landmarks between two consensus or mean configurations (Table 2). The difference vectors (blue lollipops) are presented geographically on a physical map of the New World to better illustrate the geographic distribution of the variation. The illustrated groups were selected based on the results from the hierarchical cluster analysis (see next section). As seen by the shortness of the lollipop vectors, no major shape differences were observed between the Peruvian highland sample (Cajarmarca) and Northern Chile (Fig. 2), the two coastal Peruvian samples, Ancon and MakaTampu (Fig. 2), and Northern South America and Chichen-Itza (Fig. 2) as indicated by the length and magnitude of the difference vectors (illustrated by the blue lollipops). The variation between the Patagonian and Southern Chilean samples showed the most significant shape change at the anatomical landmark bregma, which is more postero-inferiorly placed in the Chilean sample and the landmarks nasion, zygoorbitale, and zypomaxillare, which are more anteriorly located in the Chilean sample. In addition, dacryon is more supero-posteriorly placed in the Chilean sample (extreme geographic isolation can be visualized on the map). The Chichen-Itza and the Michoacán Mexican samples show biological shape differences that are illustrated by the length of the difference vectors depicted as blue lollipops (Fig. 2).

### Hierarchical Cluster Analysis

**Figure 3:**
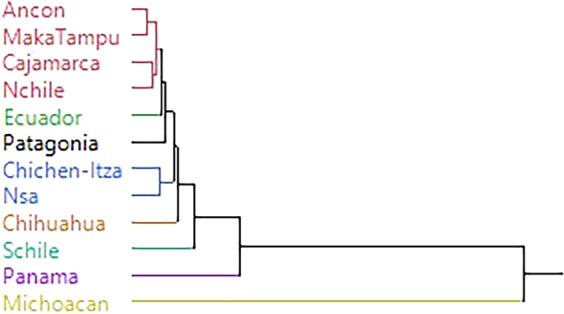
Dendrogram produced from the hierarchical cluster analysis showing the two clusters. One cluster (red) groups Peruvian coastal samples (Ancon and MakaTampu) and the two highland (Cajamarca from Peru and Northern Chile) samples. The coastal Ecuador sample and the Patagonian sample are closest to the first cluster. The second cluster (blue), groups the Chichen-Itza sample from the Yucatan and the Northern South American sample, which is comprised of combined samples from Venezuela and Colombia. All other samples are distinct with the most dissimilar being the Mexican sample from Michoacán.

**Figure 4:**
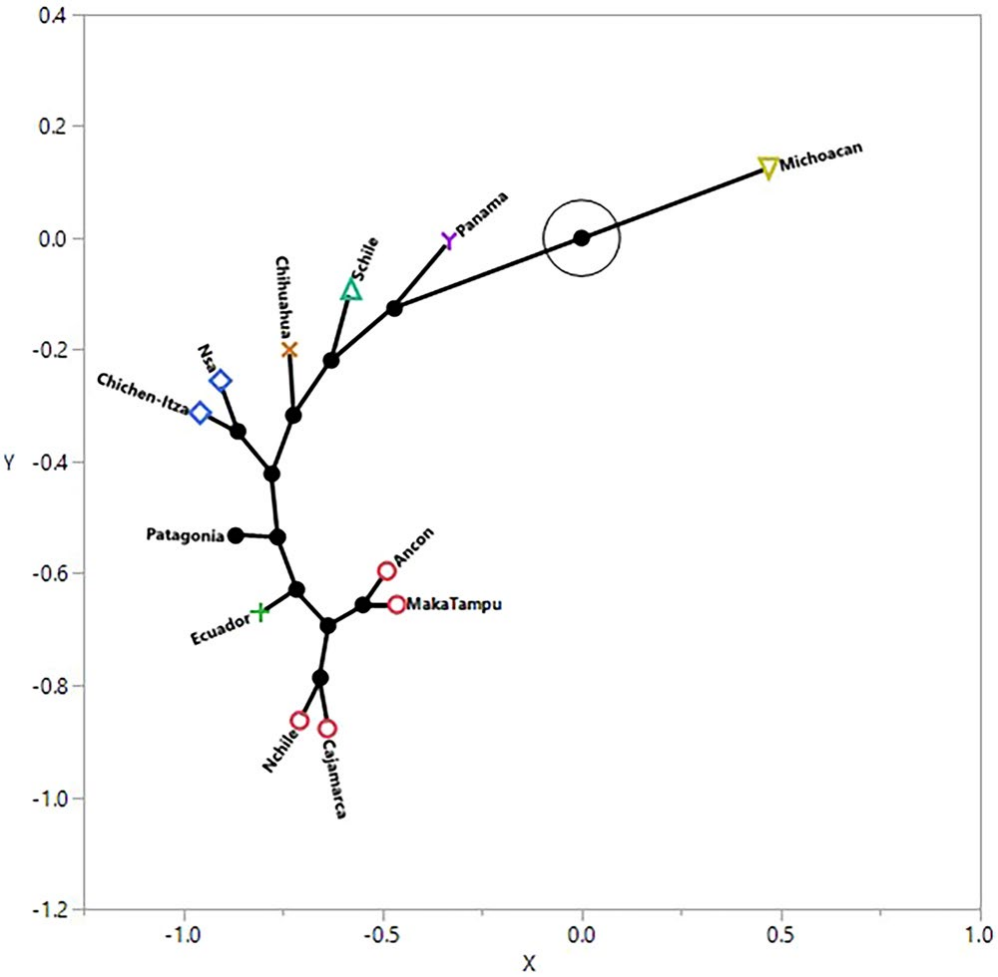
Constellation plot, which arranges the samples as endpoints, was also produced by the hierarchical cluster analysis. The length of a line between cluster joins represents the distance between the cluster joins and further illustrates the similarity between the two coastal Peruvian samples (Ancon and MakaTampu) and the two highland samples (Norther Chile and Cajamarca from Peru). It also illustrates the similarity between the Northern South America and Chichen-Itza samples (blue) with Michoacán as the most distant or dissimilar.

The dendrogram produced by the hierarchical cluster analysis using the Procrustes distance matrix shows two clusters (Fig. 3). One cluster includes the three samples from Peru and Northern Chile. Ecuador and Patagonia branch off from this cluster. Another cluster includes the Mexican sample from Chichen-Itza and Northern South America with Chihuahua branching off from this cluster. The most dissimilar is the sample from Michoacán Mexico. The constellation plot (Fig. 4) illustrates the dissimilarity of the sample from Michoacán and informs the grouping between the two coastal samples from Peru (Ancón and MakaTampu), the two highland samples (Northern Chile and Cajamarca, Peru). The similarity between Chichen-Itza and Northern South America is also illustrated. This plot clearly shows the dissimilarity of the other samples included in this study.

### Spatial analysis

Table 4 presents the spatial autocorrelation for shape (PC1 accounting for 48% of the total shape variation) and Centroid Size (CS), which shows a strong positive spatial association for morphological shape and size. The Simultaneous Spatial Autoregressive (SAR) results suggest that there is a significant effect of altitude and climate modeling craniofacial shape (F = 20.595, R^2^ = 0.723, p-value = <0.001) but not size (F = 1.184, R^2^ = 0.058, p-value = 0.308). There was a positive weak correlation for shape (PC1) and altitude (r = 0.25, p-value = <0.001) and climatic zone (r = 0.37, p-value = <0.001) but not for size (altitude r = 0.10, p-value = 0.062; climatic zone r = 0.06, p-value = 0.319).

**Table 4:** Spatial autocorrelation showing strong positive spatial association for shape and size.

Variable	Coefficient	Observed	Expected	Std Dev	Z	Pr > Z
PC1	Moran’s I	0.291	−0.00391	0.00487	60.6	<0.0001
	Geary’s c	0.215	1.00000	0.07595	−10.3	<0.0001
CS	Moran’s I	0.063	−0.00391	0.00489	13.69	<0.0001
	Geary’s c	0.868	1.00000	0.04363	−3.03	0.0024

## Discussion

Procrustes distances (Table 3) support the similarity between the Northern Chilean and Highland Peruvian samples, not entirely surprising as they are both highland groups. Earlier studies using traditional craniometrics^38,39^ demonstrated that the highland Peruvian samples showed less within-sample variation, as did the coastal samples; the coastal to highland groups displayed more between-group variation. This pattern suggests that the Andes mountain range may have acted as a barrier to gene flow, which is also evident in the morphological separation observed between the Southern Chilean sample and the Patagonian sample. The Procrustes distances show similarity between the Mexican sample (Chichen-Itza) in the Yucatan Peninsula and Central and South American samples coinciding with genetic studies showing a lack of differentiation between Mesoamerican and Andean populations^40^. The hierarchical cluster analysis links the samples from Peru to Northern Chile, with Ecuador the next closest group to these, followed by the Patagonian sample. The strong positive correlation between morphological shape and size indicates that these variables are geographically patterned. In other words, morphological form (both size and shape) in pre-contact Americas shows a clinal pattern of distribution, which supports traditional craniometric studies of the southern cone of South America^41^. The SAR analysis also suggests a significant positive weak correlation between altitude, climate, and craniofacial shape, indicating that both altitude and climate modulate craniofacial morphology. This conclusion is consistent with earlier work, which found that altitude was a factor modeling nasal shape^42^.

Was cranial morphology the result of natural selection acting on environmental and/or climatic adaptations? Or, did these arise through genetic drift (isolation-by-distance model) after initial migration? In population genetics, a central tenet when examining the spatial distribution of allele frequencies, is to first account for neutral processes such as drift^43^. The lack of consensus regarding the effects of climate on cranial traits may be related to disparate methods used to examine morphological shape, size, and form (size and shape)^42^. While some assert that size may be linked to climatic adaptation, other studies focusing on shape have a limited link to climate. Global population studies, per contra, have found that human craniofacial morphological data fit a neutral evolutionary model because contiguous populations more frequently exchange genes and/or share common ancestry^44^. In our study, we employed geometric morphometrics to examine the association between climate, altitude, and craniofacial morphology, which differs from traditional craniometrics in that shape and size can be assessed independently. The spatial autocorrelation results indicate a strong positive spatial association on morphology for both size and shape, suggesting heterogeneity of the groups as homogeneous populations usually lack spatial differentiation^43^. In addition, the SAR regression analysis also demonstrates a significant although weak effect of altitude and climate modelling shape but not size, contradicting earlier studies^45^. However, these earlier studies only examined variation at the local level and did not utilize geometric morphometric methods, which are the only undisputed approaches to separate size and shape^46^. Furthermore, the continental scale presented in this study facilitates a more complete picture of the relationship between microevolutionary forces and environmental and climatic factors. Furthermore, these data suggest that there was demic diffusion along coastal and inland routes, as evidenced by biological similarity (Table 1) between the Mexican sample from the Yucatán Peninsula and the coastal samples from South America. The earlier Peruvian analysis demonstrated the key role played by the mountainous terrain of the central Andes in producing and maintaining population differentiation (e.g., drift) supported by modern genetic studies showing little gene flow after divergence in South America^47^.

Facial morphology was not linked to ecological zones in an earlier Peruvian study, but high-altitude groups were more similar to each other, and coastal groups likewise more similar to each other, notwithstanding geographic distance^39^. The clustering of the Peruvian and Northern Chilean samples supports the closer morphological similarity between highland groups. Moreover, the extreme southern end of the South American continent is represented in this study by two samples originating from southern Chile (Chono) and the Patagonia (Rio Negro, Chubut) area of Argentina. The region represented by these samples are likewise separated from each other by the rugged Andes mountains. In consideration of the topographic features, it is not surprising that the two samples do not cluster together and are significantly different from each other. The difference vectors (Fig. 2) show that the major area of shape change is at the landmark bregma, which could be attributed to phenotypic plasticity via drift as the cranial vault is more plastic than the face or the base of the skull. The Argentine sample from Patagonia links generally with samples to the north, Northern Chile, and Peru. The southern Chile coastal sample remains distinct from the others, demonstrating the extreme isolation of this location, which is consistent with an isolation-by-distance model and possibly cold adaptation.

The effect of climatic signatures on morphological differentiation was tested on a large worldwide sample of modern humans using traditional craniometrics^43^. The neurocranium was found to be more phylogenetically recent in origin, thus, more reflective of population history than the face and other portions of the cranium that are subject to climatic selective factors. However, the basicranium is shared by both the neurocranial and facial elements with growth of each of these modules directed at maintaining and attaining stability of the whole complex^48^. Withal, each of the three major craniofacial units are driven by differential growth, development, maturation, and functional trajectories and to attain normal development each unit must maintain synchronization of the integrated systems^48^. The splanchnocranium (or neurocranium) is more susceptible to phenotypic plasticity than the phylogenetically older portions of the chondrocranium, which are thought to be more strongly genetically determined and less prone to environmental influence^48^. In a study of intentionally artificially modified and unmodified crania, landmarks that were differentiated between the culturally modified and unmodified crania were bregma and lambda on the vault, while the facial and basicranial landmarks did not differ, indicating the overall ability of the craniofacial complex as a whole to maintain stability and compensate for environmental stimuli during growth^49^. It has been proposed that significant levels of craniofacial diversification that occurred in a relatively short time span observed in southern South America cannot be fully explained by drift alone and it was concluded that random factors such as directional selection and phenotypic plasticity should also be considered^50^.

In this study, the results of the spatial autocorrelation analysis demonstrate the heterogeneity of the region that is spatially patterned consistent with an isolation-by-distance model after diffusion. In addition, there is shape-related morphological variation related to a modest contribution from altitude and climate. Drift also played a role in craniofacial diversification as observed by the differentiation of the Southern Chilean sample. Notably, this study shows that: 1. pre-contact populations in the Americas were spatially patterned; 2. Ecology and climate modelled shape-related morphology but not size-related variation; 3. And neutral evolutionary forces such as drift also patterned pre-contact populations. The results of this study generally support an isolation-by-geographic distance patterning for New World craniofacial variation, it also revealed a much more complex and multifactorial mechanism that shaped craniofacial morphology that should be considered when investigating ecogeographic models for hominin dispersals. We show that craniofacial variation is not the result of a single mechanism but is a much more complex interaction of environmental and microevolutionary forces.

## Materials and Methods

### Samples

The samples were selected for their generally excellent preservation, their representation of the distinct regions of New World and their availability at various museums. Unfortunately, little contextual information is known about the Mexican, Patagonian (from Chubut and Rio Negro Region), and Northern South American (Venezuela and Colombia) samples curated at American Museums (NMNH, AMNH, and Peabody), and what is known, such as cultural affiliation, comes from museum records. The Peruvian samples date to the Middle to Late Horizons (AD 1- 1476). The Northern Chilean sample is comprised of the Picat 8 cemetery from the Picat-Tarapacá complex dating to the Late Intermediate period (AD 800–1200). The Southern Chilean sample or Chono sample resulted from a salvage operation of skeletons from various caves and rock shelters and dates to AD 1400–1630. Undeformed crania were utilized that were sufficiently well preserved to enable the anatomical landmarks to be located and data captured. Only adult crania were included in this study and ages were assessed from the skulls at the level of adult versus juvenile. Males and females were pooled to incorporate all the observed biological variation within each sample as well as to increase sample sizes. Sex variation is negligible within each population included in population comparisons^51^. Due to sample availability and the nature of prehistoric skeletal preservation especially from tropical areas, some of the sample sizes were small. Latitude and longitude, and altitude (units in meters above sea level) were recorded based on sample locality. In addition, to examine the effect of climate on morphology each sample was scored for one of 18 global environmental zones and numerically coded using the climate map for biodiversity^52^.

Table 3 presents the sample composition, elevation, latitude and longitude, chronology, and climate. Twelve cranial samples totaling 257 individuals were examined. The geographic groups included consist of samples from Mexico, Panama, Northern and Southern Chile, Colombia, Venezuela, Ecuador, Peru, and Patagonia. The samples from Colombia and Venezuela were combined to represent one Northern South American group. Samples generally date to from the period between 0AD to 1500AD. This time period targeted Pre-Contact populations.

### Geometric morphometrics

Sixteen homologous anatomical landmarks were used in this study and are listed in Table 4. All landmarks were digitized with a Microscribe G2X ® digitizer. Because skeletal preservation is a problem in archaeological samples, data had to be imputed in some of the individuals in order to increase sample sizes and retain maximum morphological coverage but no more than 20% (or 3 landmarks) were imputed for any given individual. Data were imputed using the software Morpheus *et al*. Java Edition using the GPA mean substitution function^53^. Coordinate data must first undergo a Generalized Procrustes analysis or GPA transformation before subsequent statistical analyses can be performed. GPA translates, rotates, and scales each specimen and brings all individuals into a common coordinate system. Shape is defined as all of the geometric information that remains after the effects of location, scale, and rotational effects are removed^54,55^. Centroid size is a measure of geometric scale that is mathematically independent of shape^56^. The GPA procedure was performed using MorphoJ, which is freely available for downloading and developed by Klingenberg^54^. A principal component analysis (PCA) of the covariance matrix was conducted on the GPA transformed coordinates to reduce dimensionality of the data for subsequent multivariate statistical analyses^54^.

To examine shape and size (e.g., Centroid Size) variation among the groups, a Procrustes ANOVA was performed using principal component scores calculated from the PCA^54^. A canonical variates analysis (CVA) was performed to account for the maximum amount of among-group variance relative to within-group variance, which are also uncorrelated within and among groups. CVA is used to examine variation for more than two groups known *a priori* that presents the most variation with the least dimensions possible^54^. A Procrustes distance, which measures the distance between the individuals/landmark configurations was used to examine group variation^56^. An average linkage hierarchical (or agglomerative) cluster analyses was performed using the Procrustes distance matrix to examine group similarity. Hierarchical clustering begins with every sample in a single cluster, then in each successive iteration, it merges the closest pair of clusters (distances between all pairs and averages all these distances) until all the data are in one cluster. The cluster analysis was performed in JMP ® Pro 14^57^.

A discriminant function analysis (DFA) using crossvalidation was carried out for each group. Between-group variation was depicted via difference vectors that illustrate the magnitude and direction of shape differences between two group means or consensus configurations. These analyses were performed using the software program MorphoJ, Apache License Version 2.0^54^.

### Spatial analysis

Spatial autocorrelation was estimated using the principal components calculated in the geometric morphometric analysis (Morans’s I, a product-moment coefficient, and Geary’s c, a distance-type coefficient) to measure the distribution of spatial feature locations (latitude/longitude) and the variables cluster or disperse. The autocorrelation coefficient r is a measure of the genetic similarity between individuals with reference to geographic separation^40^, which was performed using the variogram procedure in SAS 9.4^58^. The variogram procedure reports Moran’s I and Geary’s c as a standardized Z-score. A positive autocorrelation is indicated when ZI >0 and Zc <0 and a negative correlation is expressed when ZI <0 and Zc >0^58^. To examine the relationship between altitude and morphology a Simultaneous Spatial Autoregressive (SAR) was used, which is commonly used in spatial ecological analysis that incorporates autocorrelation into residuals^59^. R^2^ provides a measure of fit of the data to the model as the proportion of the total variation being explained^4^. A perfect fit between the data and regression line is denoted by an R^2^ of 1 or −1, values around 0 signify there is no association between the variables (response variables = PC1 and Centroid Size; predictor variables = altitude and bioclimatic zones)^4^. The elevation between the present-day political borders were used for Venezuela and Colombia as the two samples were combined. To evaluate the relative contributions of geographical location, altitude and climate on morphology, inter-correlations considering the spatial structure were calculated. The SAR analysis was performed using the SAM-Spatial Analysis in Macroecology software 4.0^59^. Spatial regression techniques have been found to be provide more accurate estimates of the association between morphological and ecological variables than the Mantel test^60^.

## Supplementary information

Dataset 1

## Data Availability

Raw coordinate data (with accession codes) are available in the online version of this work.
